# Gamma background measurements in the Gran Sasso National Laboratory

**DOI:** 10.1007/s10967-012-1990-9

**Published:** 2012-07-25

**Authors:** Dariusz Malczewski, Jan Kisiel, Jerzy Dorda

**Affiliations:** 1 Faculty of Earth Sciences, University of Silesia, Bedzinska 60, 41-200 Sosnowiec, Poland; 2 Institute of Physics, University of Silesia, Uniwersytecka 4, 40-007 Katowice, Poland

**Keywords:** Gamma-ray spectrometry, Gamma background, Gamma fluxes, Underground laboratory

## Abstract

In situ gamma-ray measurements were taken at eight locations in the Gran Sasso National Laboratory (Italy). Count rates for gamma radiation within the energy range of 7–2,734 keV varied from 8 to 60 γ s^−1^. The arithmetic mean was 49 γ s^−1^ for measurements taken without a collimator. The average gamma flux inside the Lab was 0.25 γ cm^−2^ s^−1^. The sedimentary rocks surrounding the Lab are characterized by low activity concentrations of uranium and thorium, equal to 1.7 and 1.4 Bq kg^−1^, respectively.

## Introduction

The Gran Sasso National Laboratory (Laboratori Nazionali del Gran Sasso—LNGS) is one of the largest and most important underground research centers in the world. The Laboratory is composed of three large underground halls that have been excavated in the Gran Sasso Massif (central Italy). Including connecting tunnels and emergency passages, the total volume and area of the Lab are 1.8 × 10^5^ m^3^ and 1.35 × 10^4^ m^2^, respectively [1]. The scientific programs at LNGS comprise nuclear physics, elementary particle physics, astrophysics, and dark matter detection, and their sensitive experiments require an environment shielded from high energy cosmic rays [2]. As the Laboratory is deep underground, a thick layer of overlaying rocks effectively blocks the strongly interacting cosmic rays from entering the underground halls where the experiments are run. After cosmic rays, the most important sources of background radiation include the decay of primordial radionuclides, such as ^40^K, ^232^Th, and ^238^U in rock, concrete, and the construction materials used in the detectors. The neutrons originating from (α, n) reactions and the spontaneous fission of U and Th are the main sources of background radiation, which can imitate some of the expected signals from physics experiments [3, 4].

In this paper we present results of in situ gamma-ray measurements in the LNGS, which were performed in 2007, and laboratory measurements of the parent rock and concrete covering the tunnels.

### Location of in situ measurements

**Fig. 1:**
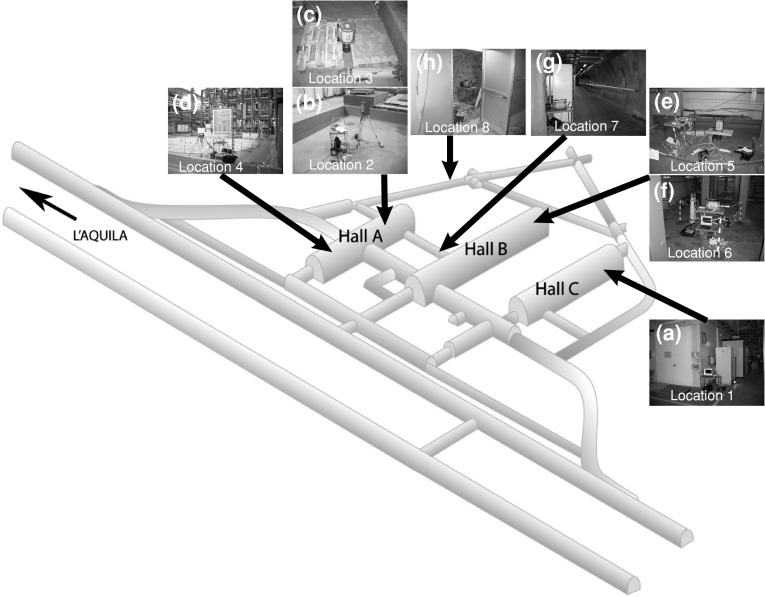
Location of in situ measurements (overall view of the Lab by the kind permission of the Public Affairs Office of the Gran Sasso National Laboratory)

**Fig. 2:**
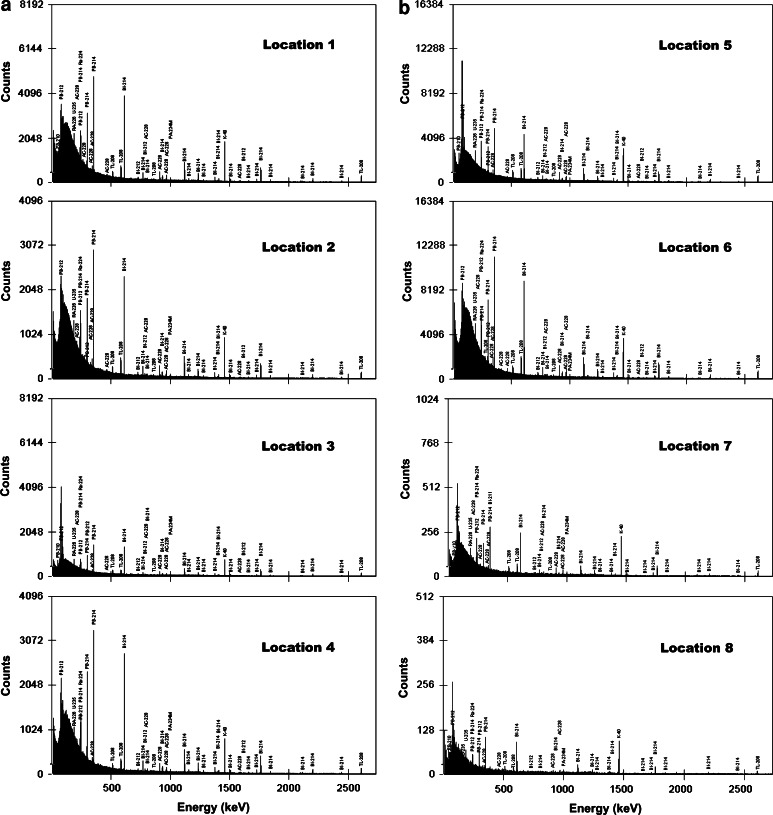
In situ gamma-ray spectra at locations: (**a**) 1, 2, 3 and 4 (**b**) 5, 6, 7 and 8. The characteristic gamma-ray emitters are marked above the corresponding peaks

Measurement location 1 was chosen at the back of Hall C, and was 3.2 m from the Borexino experiment [5]. The end cap of the detector was 90 cm above the concrete base (Fig. 1a). Measurement location 2 was located in Hall A. The detector was mounted 90 cm above the concrete floor opposite to the LVD experiment [6] (Fig. 1b). The third location was the same as the second, except that the detector was placed directly above the concrete surface (Fig. 1c). Location 4 was also located in Hall A, close to an empty metal place and 16 m away from the LVD construction. The detector was placed 70 cm above the concrete base (Fig. 1d). Measurement location 5 was located in Hall B at the back of the ICARUS experiment [7]. The measurement was performed with a collimator directly above the concrete surface (Fig. 1e). Location 6 was the same as 5, except that the detector was mounted horizontally 14 m from the ICARUS construction and 90 cm above the concrete base (Fig. 1f). Measurement location 7 was located in the side tunnel that connects Halls A and B. The measurement at this location used a collimator directly by the tunnel wall and 90 cm above the ground (Fig. 1g). The last measuring point (location 8) was located in a small cavern in dolomitic limestone, which is the parent rock from which the Lab was excavated [8]. The detector with a collimator was mounted horizontally directly near the rock and 90 cm above the ground (Fig. 1h). The gamma-ray spectra from all locations are presented in Fig. 2.

### Materials and methods

The background gamma radiation in LNGS was measured in situ using a portable gamma-ray spectrometry workstation (Fig. 1). The GX3020 system consisted of a coaxial HPGe detector (32 % efficiency, crystal length 59 mm, and diameter 56.6 mm) with a cryostat mounted on a tripod or a special table, a collimator 50 × 180 mm with diameter 80 mm, a multichannel buffer (InSpector 2000 DSP), and a laptop. The detector bias voltage was 4,000 V and the energy resolution was 0.8 keV at 122 keV and 1.7 keV at 1.33 MeV. Three software packages were used for the efficiency calibration and the determination of radionuclides: In Situ Object Counting Software (ISOCS), Laboratory Sourceless Calibration Software (LabSOCS), and Genie 2000 v.3. The total duration of a single measurement varied from about 2 h to 65 h (Table 1). The use of a collimator allowed us to determine an average gamma flux (expressed in γ cm^−2^ s^−1^) in the LNGS.

**Table 1:** Count rates (γ s^−1^) in specified energy ranges. Measurements at locations 3, 5, 7, and 8 were performed using a collimator

Location	7.4–2,734.2 keV	7.4–249.8 keV	250.2–500.4 keV	500.8–1,005.2 keV	1,005.6–1,555.8 keV	1,556.2–2,055.8 keV	2,056.2–2,734.2 keV
**1** (60418)^a^	34.70(2)	22.66	6.22	3.59	1.66	0.38	0.21
**2** (23138)	52.05(5)	35.50	8.64	5.00	2.28	0.54	0.29
**3** (61314)	11.57(1)	6.87	2.15	1.53	0.75	0.18	0.10
**4** (17569)	60.09(6)	39.32	10.74	6.18	2.81	0.70	0.34
**5** (235489)	11.54(1)	6.89	2.13	1.50	0.74	0.18	0.10
**6** (100698)	47.84(2)	32.20	8.08	4.68	2.08	0.51	0.30
**7** (7344)	19.32(5)	11.99	3.34	2.36	1.23	0.26	0.15
**8** (7457)	7.60(3)	5.06	1.18	0.81	0.43	0.084	0.052

^a^Measurement time (s)

Samples of dolomitic limestone and concrete from measurement locations 7 and 8 were crushed and dried, and several months after collection they were placed in a Marinelli beaker with volume 450 cm^3^, and measured using the same GX3020 HPGe detector in a lead and copper shield (60 mm). The spectrometer energy was calibrated using homogeneously dispersed ^241^Am, ^109^Cd, ^139^Ce, ^57^Co, ^60^Co, ^137^Cs, ^113^Sn, ^85^Sr, ^88^Y, and ^203^Hg radioisotopes in a silicone resin [certificate source type Marinelli Beaker Standard Source (MBSS), supplied by the Czech Metrological Institute]. The measurements were done at the Laboratory of Natural Radioactivity (Faculty of Earth Sciences, University of Silesia).

## Results and discussion

The count rates (γ s^−1^) at all measurement locations are listed in Table 1, and the gamma-ray fluxes in γ cm^−2^ s^−1^ from locations 3, 5, 7, and 8 are provided in Table 2. The count rates in the main gamma peaks and the gamma fluxes from these peaks at locations 3, 5, 7, and 8 are presented in Tables 3 and 4, respectively. Table 5 summarizes the results of the activity measurements in the parent rock and in concrete fragments from the side tunnel connecting Halls A and B.

**Table 2:** Gamma fluxes in γ cm^−2^ s^−1^ in specified energy ranges, at locations 3, 5, 7, and 8

Location	7.4–2,734.2 keV	7.4–249.8 keV	250.2–500.4 keV	500.8–1,005.2 keV	1,005.6–1,555.8 keV	1,556.2–2,055.8 keV	2,056.2–2,734.2 keV
**3**	0.230	0.137	4.27 × 10^−2^	3.04 × 10^−2^	1.49 × 10^−2^	3.61 × 10^−3^	1.90 × 10^−3^
**5**	0.230	0.137	4.24 × 10^−2^	2.99 × 10^−2^	1.46 × 10^−2^	3.50 × 10^−3^	2.02 × 10^−3^
**7**	0.384	0.239	6.63 × 10^−2^	4.69 × 10^−2^	2.45 × 10^−2^	5.11 × 10^−3^	2.95 × 10^−3^
**8**	0.151	0.101	2.34 × 10^−2^	1.60 × 10^−2^	8.48 × 10^−3^	1.66 × 10^−3^	1.03 × 10^−3^

**Table 3:** Count rates (γ s^−1^) in the main gamma peaks. Measurements at locations 3, 5, 7, and 8 were performed using a collimator

Location	351.9 keV ^214^Pb (^238^U) 1.27 keV ^a^	609.3 keV ^214^Bi (^238^U) 1.41 keV	911.6 keV ^228^Ac (^232^Th) 1.61 keV	1,460.8 keV ^40^K 1.91 keV	2,204.2 keV ^214^Bi (^238^U) 2.17 keV	2,614.5 keV ^208^Tl (^232^Th) 2.44 keV
**1**	0.425^b^	0.332	0.060	0.195	0.022	0.036
**2**	0.590	0.507	0.091	0.261	0.029	0.050
**3**	0.125	0.110	0.022	0.081	0.011	0.016
**4**	0.936	0.768	0.096	0.290	0.042	0.056
**5**	0.120	0.097	0.026	0.084	0.009	0.019
**6**	0.577	0.450	0.089	0.230	0.030	0.056
**7**	0.206	0.176	0.041	0.188	0.012	0.028
**8**	0.069	0.073	0.012	0.060	0.004	0.010

^a^Full width at half maximum (FWHM)

^b^Estimated uncertainty of peak areas ≤5 %

**Table 4:** Gamma fluxes in γ cm^−2^ s^−1^ from the main gamma peaks, at locations 3, 5, 7, and 8

Location	351.9 keV ^214^Pb (^238^U) 1.27 keV ^a^	609.3 keV ^214^Bi (^238^U) 1.41 keV	911.6 keV ^228^Ac (^232^Th) 1.61 keV	1,460.8 keV ^40^K 1.91 keV	2,204.2 keV ^214^Bi (^238^U) 2.17 keV	2,614.5 keV ^208^Tl (^232^Th) 2.44 keV
**3**	2.49 × 10^−3^ ^b^	2.18 × 10^−3^	4.34 × 10^−4^	1.60 × 10^−3^	2.15 × 10^−4^	3.14 × 10^−4^
**5**	2.39 × 10^−3^	1.94 × 10^−3^	5.20 × 10^−4^	1.66 × 10^−3^	1.82 × 10^−4^	3.69 × 10^−4^
**7**	4.09 × 10^−3^	3.51 × 10^−3^	8.21 × 10^−4^	3.74 × 10^−3^	2.30 × 10^−4^	5.53 × 10^−4^
**8**	1.37 × 10^−3^	1.46 × 10^−3^	2.43 × 10^−4^	1.19 × 10^−3^	7.47 × 10^−5^	1.89 × 10^−4^

^a^Full width at half maximum (FWHM)

^b^Estimated uncertainty of peak areas ≤5 %

**Table 5:** Dolomitic limestone and concrete ^40^K, ^232^Th, and ^238^U concentrations

^40^K (Bq kg^−1^)	^232^Th (Bq kg^−1^)	^238^U (Bq kg^−1^)
Dolomitic limestone (parent rock)		
26(2)	1.5(1)	1.8(1)

^40^K (10^−6^ g g^−1^)	^232^Th (10^−6^ g g^−1^)	^238^U (10^−6^ g g^−1^)
Dolomitic limestone (parent rock)		
0.10(1)	0.36(2)	0.14(1)

^40^K (Bq kg^−1^)	^232^Th (Bq kg^−1^)	^238^U (Bq kg^−1^)
Concrete		
70(2)	3.7(2)	9.5(3)

^40^K (10^−6^ g g^−1^)	^232^Th (10^−6^ g g^−1^)	^238^U (10^−6^ g g^−1^)
Concrete		
0.27(1)	0.90(5)	0.76(2)

**Fig. 3:**
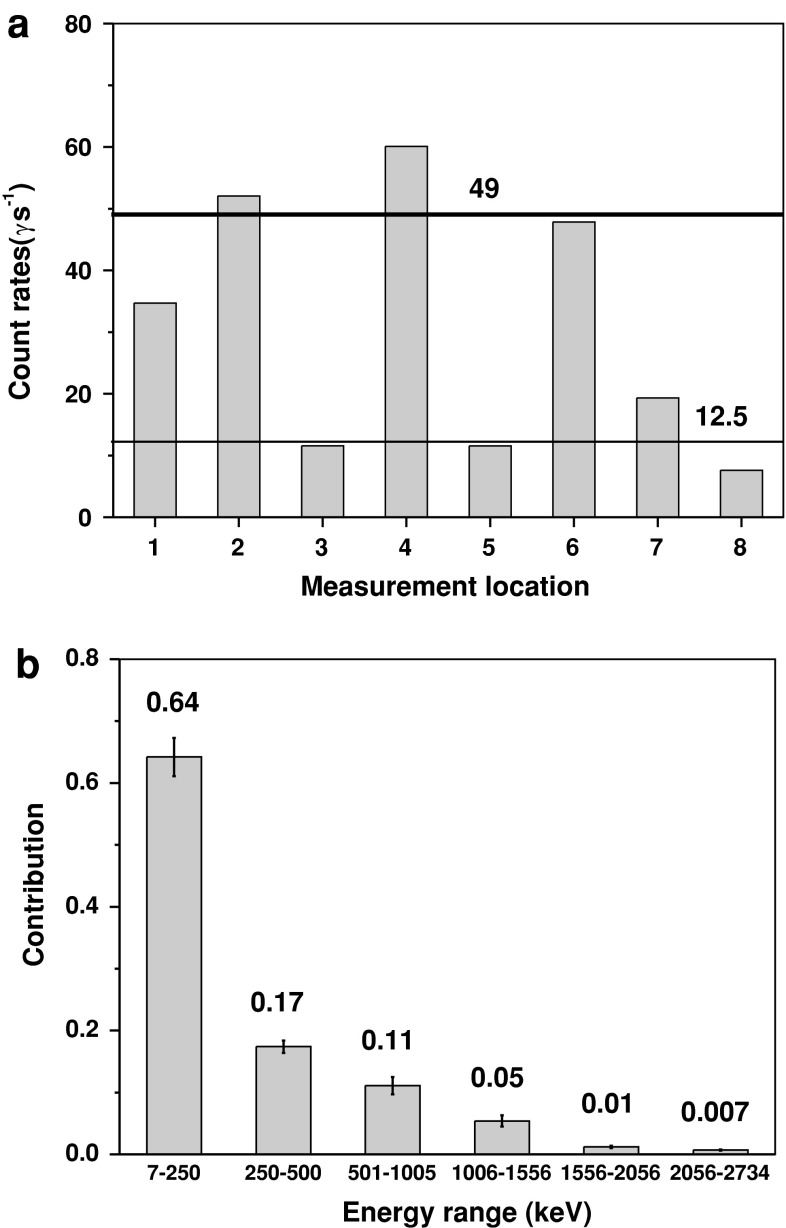
**a** Count rates at all locations. *Thick solid line* an average count rate from measurements without a collimator (49); *thin solid line* an average count rate from measurements with a collimator (13). **b** Average contributions of count rates within the particular energy ranges from all locations

The total count rates in the energy range 7.4–2,734.2 keV varied from 7.6 γ s^−1^ at location 8 to ~60.1 γ s^−1^ at location 4 (Table 1). The arithmetic means for the measurements without a collimator (locations 1, 2, 4, and 6) and with a collimator (locations 3, 5, 7, and 8) were 49(9) and 13(4) γ s^−1^, respectively (Fig. 3a). As expected, the highest count rates were noted at energies ranging between 7.4 and 249.8 keV, with an average contribution of 0.64 for all measurement locations (Fig. 3b). The count rates at subsequent energy ranges noticeably decreased, with average contributions of 0.17, 0.11, 0.05, 1.2 × 10^−3^, and 7 × 10^−3^ within ranges 250–500, 501–1,005, 1,006–1,556, 1,556–2,056, and 2,056–2,734 keV, respectively (Table 1; Fig. 3b).

For measurements using the collimator (locations 3, 5, 7, and 8), the highest total gamma flux, 0.384 γ cm^−2^ s^−1^, was noted at location 7 located directly in front of the concrete-covered walls of the side tunnel (Table 2; Fig. 1g). The same total gamma flux, 0.230 γ cm^−2^ s^−1^, was observed at points 3 and 5 located just above the concrete surface (Table 2; Fig. 1c, e). The lowest total gamma flux, 0.151 γ cm^−2^ s^−1^, was observed at point 8 located right in front of the parent rock (Fig. 1h). Similar to the count rates, the particular contributions of these gamma fluxes rapidly decrease with increasing energy values; they are on the order of 10^−2^ γ cm^−2^ s^−1^ in the range 250–1,556 keV, and 10^−3^ γ cm^−2^ s^−1^ in the range 1,556–2,734 keV.

The highest integral areas from the main gamma transitions were noted under the peak at 351.9 keV (^214^Pb), and then under the peaks at 609.3 keV (^214^Bi) and 1,460.8 keV (^40^K) (Table 3). The two most intense gamma transitions from the ^232^Th series, i.e., 911.6 keV (^228^Ac) and 2,614.5 keV (^208^Tl), are characterized by reduced areas compared with lines 351.9, 609.3, and 1,460.8 keV. The gamma transition of 2,204.2 keV from ^214^Bi (^238^U) is characterized by the lowest count rates, ranging from 0.022 γ s^−1^ at location 1 to 0.042 γ s^−1^ at location 4 (measurements without a collimator), and from 0.004 γ s^−1^ at location 8 to 0.012 γ s^−1^ at location 7 (measurements with a collimator). Despite the low yield (1.28 %) of the 2,204.2 keV transition [9], its contribution may be important to geo-neutrino experiments because of its possible overlap with the deuteron binding energy of 2.2 MeV. This energy is released as gamma rays as a result of inverse beta decay in a liquid scintillator [10, 11].

Gamma fluxes at points 3, 5, 7, and 8 (measurements with a collimator) are on the order of 10^−3^ γ cm^−2^ s^−1^ for the peaks at 351.9, 609.3, and 1,460.8 keV, and on the order of 10^−4^ γ cm^−2^ s^−1^ for the peaks at 911.6, 2,204.2, and 2,614.5 keV (Table 4).

The results of the activity measurements for dolomitic limestone and concrete collected from locations 7 and 8 are provided in Table 5. Values similar to those presented here have been reported in previous measurements [3, 12]. As seen in the table, the activity concentrations in Bq kg^−1^ and the concentrations in 10^−6^ g g^−1^ of ^40^K and ^232^Th in concrete are about three times higher than those in dolomitic limestone. The concentration of ^238^U is about five times higher in concrete than in dolomitic limestone. Activity concentrations of ^40^K, ^232^Th, and ^238^U equal to 70, 8, and 25 Bq kg^−1^, respectively, have been noted in typical carbonate rocks, and concentrations equal to 89, 8.5, and 31 Bq kg^−1^, respectively, have been noted in limestone concretes [13]. Both the dolomitic limestone and the concrete covering tunnels in LNGS have concentrations of ^40^K and ^232^Th that are clearly below the levels observed for typical carbonate rocks and limestone concrete. The ^40^K concentrations are roughly comparable, but the ^40^K activity in the dolomite limestone at location 8 is still three times lower than the activity for typical carbonate rocks.

## Conclusions

The average measured gamma background in LNGS was 49 γ s^−1^ in the energy range of 7.4–2,734.2 keV. Gamma fluxes were obtained from four locations for parent rock and concrete. The gamma flux for the parent rock was 0.151 γ cm^−2^ s^−1^, and the flux values for the concrete varied between 0.230 and 0.384 γ cm^−2^ s^−1^. The average value for both the parent rock and concrete was 0.249 γ cm^−2^ s^−1^. Samples of both dolomite limestone and concrete showed low activity concentrations of ^40^K, ^232^Th, and ^238^U, which are below the activity concentrations of these primordial radionuclides as noted in typical carbonate rocks and limestone concrete.
